# The Silent Transformation of Stereotactic Brain Biopsies After the Introduction of Robotics

**DOI:** 10.1002/rcs.70087

**Published:** 2025-07-05

**Authors:** Eliane Weinbrenner, Mykola Gorbachuk, Kathrin Machetanz, Florian Grimm, Linda Oberle, Sophie S. Wang, Marcos Tatagiba, Georgios Naros

**Affiliations:** ^1^ Department of Neurosurgery and Neurotechnology Eberhard Karls University Tuebingen Germany

**Keywords:** brain biopsy, frame‐less stereotactic surgery, robot‐assisted surgery, robotics, stereotaxy

## Abstract

**Background:**

In frame‐based stereotaxy, the design of the frame limits trajectory selection, e.g., to the temporal lobe and posterior fossa. We hypothesise that frame‐less neuronavigation and robotic technology might have expanded these stereotactic corridors.

**Methods:**

We analysed 376 frame‐based, neuronavigated and robotic brain biopsies. We analysed entry (EP) and target (TP) point coordinates, trajectory lengths (TL) and angles (*α*_1_,*α*_2_), skin‐to‐skin time (STS), diagnostic yield and morbidity.

**Results:**

Robotics liberated *TP* and *EP* selection, enabling trajectories not applicable with the frame. There was an increased application of lateral trajectories (reducing *α1*) while decreasing *TL*. There was a significant *STS* reduction attributable to a modification of the surgical approach (twist drill vs. burr hole).

**Conclusions:**

Robotics modified trajectory selection and the surgical approach. Duration and invasiveness of brain biopsies were decreased without affecting diagnostic yield or morbidity. This may represent a clinical benefit of robotics compared with frame‐based and frame‐less stereotaxy.

**Abbreviations**
BFR: bone fiducial registration
CT: computer tomography
DY: diagnostic yield
EP: entry point
GTS: distance between the guiding tube and the skull
HGG: high grade glioma
LGG: low grade glioma
LSR: laser surface registration
MRI: magnetic resonance imaging
PM: permanent morbidity
SA: surgical approach
SEEG: stereoelectroencephalography
STS: skin‐to‐skin time
TL: trajectory length
TP: target point

## 1 Introduction

More than 70 years after its introduction [1], frame‐based stereotaxy remains the gold standard for precise targeting within the brain [2, 3, 4, 5]. Conventional stereotaxy enables targeting within the brain depending on three‐dimensional coordinates in relation to an external frame‐based origin. Since then, several stereotactic frames have been developed, different in their design but remaining faithful to the original concept [2, 3].

In contrast, trajectory planning has changed substantially in conjunction with the technical evolution in brain imaging. In the early years of stereotaxy, the entry (EP) and target (TP) points of trajectories were usually selected in horizontal and vertical planes to minimise the length of the trajectory [6]. With the introduction of computer tomography (CT) and magnetic resonance (MR) imaging, neurosurgeons gain information about the neurovascular anatomy along the stereotactic trajectory. Thus, trajectories were modified to avoid critical structures and to reduce complication rates [7]. Against this background, a few basic principles in trajectory planning have evolved, for example, avoiding sulci, transventricular trajectories and the unnecessary violation of eloquent areas [2, 3, 7, 8]. In conventional stereotaxy, however, the design of the frame and patient's positioning need to be considered as rods and bars are limiting trajectory selection [3, 9]. In particular, entry points at the lateral or dorsal aspect of the skull are usually inaccessible [10]. Furthermore, a supine positioning and frontal entry points are recommended to avoid CSF loss and brain shift [11, 12]. In subliminal consideration of these principles, most frame‐based procedures are usually performed following a few stereotactic corridors. In frame‐less stereotaxy (i.e., robotics, neuronavigation), the omission of the frame provides a technical advantage enabling additional trajectories potentially blurring the conventional stereotactic corridors [3, 13]. In line, recent publications have documented the advantage of robotics in the application of cerebellar biopsies and *trans*‐cerebellar trajectories to the brainstem, which are hardly accessible by conventional stereotaxy [10].

The aim of the present study was to evaluate whether the way stereotactic biopsies are obtained has changed since the introduction of frame‐less robotics in comparison to conventional frame‐based or frame‐less stereotaxy.

## 2 Materials and Method

### 2.1 Patients

This retrospective study enroled 376 stereotactic trajectories in 358 consecutive patients undergoing brain biopsy between 03/2009 and 11/2021. In this period, three different robot‐assisted (*ROBOT*) and non‐robotic (*NON‐ROBOT*) technologies have been used to acquire samples. Non‐robotic surgeries were based on frame‐based (*FRAME*) and frame‐less/neuronavigated (*NAVIGATION*) techniques. Decisions for biopsy were made during institutional multidisciplinary patient management conferences. The choice of the surgical procedure was based on the availability of the respective systems. The study was approved by the local ethics committee (No. 015/2020BO2) and performed in accordance with the Declaration of Helsinki. As the analysis was retrospective and utilised anonymised data originally collected for clinical purposes, obtaining explicit patient consent was not required. The inclusion criteria were (i) a lesion affecting the brain, cerebellum, or brain stem, (ii) the presence of a preoperative high resolution MR imaging and (iii) the presence of a postoperative CT or MR imaging. The results are reported following the STROBE guidelines [14].

### 2.2 Standard Operating Procedure (SOP) for Intracranial Biopsies

Patients usually receive a preoperative high resolution T1‐weighted (double) contrast‐enhanced MPRAGE sequence (slice thickness: 1.0 mm; TR: 3200 ms; TE: 381 ms; TI: 1100; flip angle: 8°; pixel bandwidth: 130; pixel spacing: 1/1 mm; matrix: 256x256). These sequences were imported into the planning software of BrainLab (iPlanet 3.1, Brainlab, Munich, Germany) or the ROSA robot (ROSA One, Zimmer Biomet, Warsaw, USA). The EP and TP of the stereotactic trajectory were planned following general stereotactic principles: (i) avoiding sulci or transventricular trajectories and the unnecessary violation of eloquent areas, (ii) avoiding conflicts with the stereotactic frame (*FRAME*), and (iii) shortening the trajectory lengths [2, 3, 7, 8].

**FIGURE 1:**
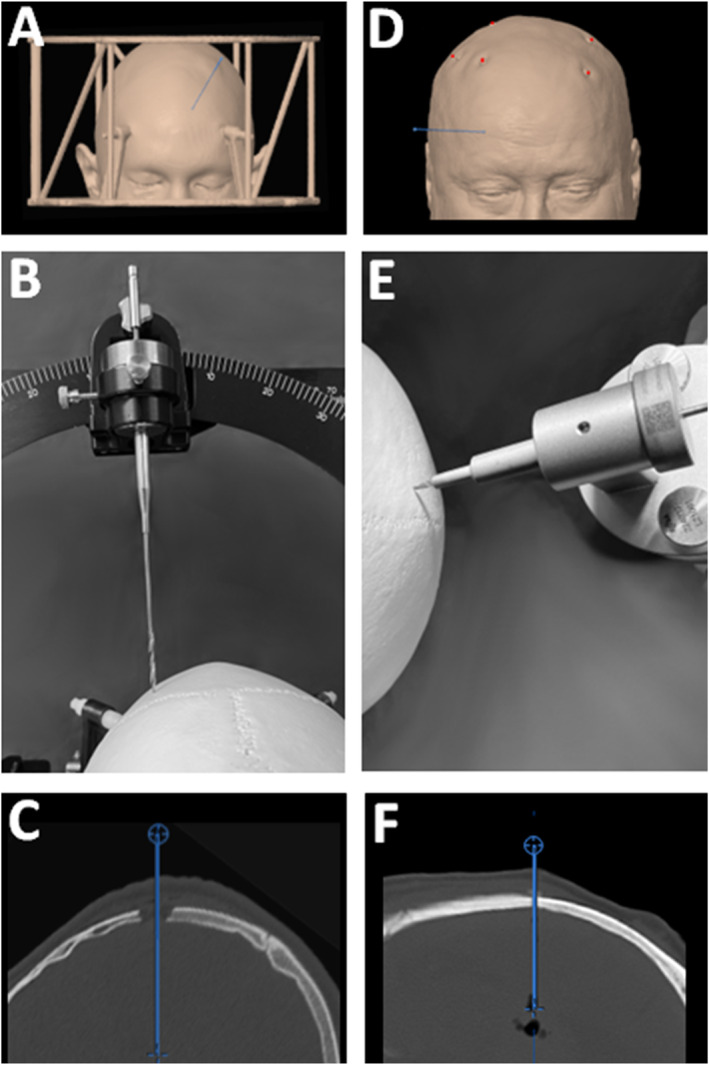
Surgical approaches in frame‐based and robot‐assisted stereotaxy. Stereotactic frames affect the trajectory selection (particular with lateral and dorsal entry points) due to their design (e.g., rods) (A). Furthermore, twist drill approaches are unfavoured in frame‐based stereotaxy due to the risk of slipping of the drill along the skull in tangential trajectories (B). Instead, burr hole approaches are preferred (C). Robot‐assisted frame‐less stereotaxy is based on surface‐ or bone fiducial‐based registration techniques (D), bone fiducials indicated by red dots. By the active reduction of the distance between the guiding tube of the robot and the skull during surgery, twist drill accuracy is facilitated and assured (E and F).

In frame‐based stereotaxy (*FRAME*), the stereotactic frame (CRW Radionics Inc., Burlington, Massachusetts, USA) was usually applied at the bedside. A preoperative CT scan (1 mm isovoxel, contrast‐enhanced multi‐slice CT scanner, Siemens Medical GmbH) was acquired with a localiser fixed to the stereotactic frame (Figure 1). All robot‐assisted biopsies were performed using the ROSA one robot (Zimmer‐Biomet, Warsaw, USA). Patient‐to‐robot registration is performed either by (i) pair‐point registration (e.g., bone fiducial registration, *BFR*) or (ii) laser surface registration (*LSR*). For *BFR*, five bone (WayPoint, FHC, Bowdoin, USA) fiducials were placed on the day of surgery at the bedside under local anaesthesia just before preoperative CT scanning (Figure 1). All *NAVIGATION* biopsies were performed with the assistance of the VarioGuide system (Brainlab, Munich, Germany). This system enables both BFR and LSR registration (see above). The planning software (iPlanet 3.1, Brainlab, Munich, Germany) provided the coordinates, which were manually applied to the VarioGuide system.

The subsequent intraoperative steps were equal for both the *NON‐ROBOT and ROBOT* groups and performed under general anaesthesia. The patient's head was fixed by the stereotactic frame (*FRAME*) or by a Mayfield skull clamp (*NAVIGATION* and *ROBOT*). Depending on the trajectory characteristics, surgeons decided on the surgical approach (*SA*), that is, conventional burr hole (*BH*) or a twist drill (*TD*). In the *BH* approach, a 3 cm skin incision was performed followed by the 1.4 cm burr hole trepanation (Elan 4, B. Braun, Freiburg, Germany). For the *TD* approach, there was a simple stab incision (approx. 2 mm) of the skin. Trepanation was performed using a 2.1 mm twist drill (Acculan 4, B. Braun, Freiburg, Germany). Major differences are notable in the guidance of the twist drill (Figure 1). In both groups, biopsies were taken in several quadrants along the trajectory with a biopsy cannula (1.8 × 250 mm, BrainPro, Pajunk, Geisingen, Germany). Frozen section examination was performed. A postoperative CT scan was performed in each patient within 24 h after the surgery to exclude any operative complication and to verify the target.

### 2.3 Clinical Data

We analysed the skin‐to‐skin (*STS*) time as a surrogate parameter for the time efficiency of frame‐based or robot‐assisted biopsies. Rate of symptomatic hemorrhages and permanent morbidity were systematically analysed. Histological results were collected to evaluate the diagnostic yield (*DY*).

### 2.4 Trajectory Characteristics

Matlab (R2019a, Mathworks Inc., Natick, MA, USA) and the SPM 12 toolbox [15] were used for systematic evaluation and visualisation of the position and length of trajectories. *EP* and *TP* coordinates were acquired from the planning software. *EP* and *TP* of trajectories were imprinted into the pre‐operative MRI using custom‐based MATLAB scripts. Preoperative MR images were spatially normalised into the *MNI* space using SPM 12 [15]. This approach enables visualisation of different patients in a common space and compares *EP* and *TP* coordinates. Normalised (*X,Y,Z*) coordinates of the *EP* and *TP* were acquired and used to calculate the length of the trajectory (*TL*) as well as the trajectory angles about the y‐ (*α1*) and *x*‐axis (*α2*). We compared the normalised *TP* coordinates with the *AAL* atlas [16] to classify the anatomical *TP* location. This classification was visually reviewed and corrected for regions unrecognised by the *AAL* atlas (e.g., white matter tracts, brainstem, cerebellum). Subsequently, we evaluated *EP* and trajectory characteristics depending on the anatomical *TP* location.

### 2.5 Statistical Analysis

All statistical tests were performed using Matlab (R2019a, Mathworks Inc., Natick, MA, USA) and SPSS (IBM SPSS Statistics for Windows, version 22.0. Armonk, NY: IBM Corp.). Group differences in categorical variables (e.g., gender, histological results, etc.) were compared using X^2^‐test with Yates' correction. Metric variables (e.g., age, STS times, coordinates, trajectory lengths and angles) were evaluated with a Kruskal–Wallis test. The effect of several independent variables (e.g., *GROUP* and *SA*) on a single dependent variable (e.g., *STS*) was evaluated by a two‐way analysis of variance (*ANOVA*). Data are shown as mean ± standard deviation (SD). *p*‐values < 0.05 were considered significant.

## 3 Results

### 3.1 Clinical Characteristics

This retrospective study evaluated 376 stereotactic trajectories in 358 patients undergoing conventional frame‐based, frameless/neuronavigated, or robot‐assisted brain biopsies. Notably, due to the design of the frame, no (*trans*‐) cerebellar biopsies were performed in the *FRAME* group. The application of the robotic technology significantly reduced the operation time, halving the skin‐to‐skin time. All techniques exerted an excellent diagnostic yield. There was no significant difference in the occurrence of complications. The overall risk of symptomatic haemorrhage was 9/358 (2.5%), necessitating a revision surgery in 4/358 (1.1%). The risk of permanent neurological deterioration was 5/358 (1.4%). There were no significant group differences (Table 1).

**TABLE 1:** Patients' characteristics.

	Frame	Robot	Navigation
No. of patients	177	150	31
Age (mean ± SD) Gender (f:m)	58.5 ± 17.7 82:95	56.6 ± 21.8 63:87	66.6 ± 13.9 12:19
Trajectories number Target locations frontal Parietal Temporal Occipital Insula cerebellum Brainstem Basal ganglia^a^ misc.	177 59 (33.3%) 22 (12.4%) 16 (9.0%) 3 (1.7%) 11 (6.2%) 0 (0%) 4 (2.3%) 60 (33.9%) 2 (1.1%)	168 38 (22.6%) 25 (14.9%) 24 (14.3%) 5 (3.0%) 7 (4.2%) 28 (16.7%) 8 (4.8%) 30 (17.9%) 3 (1.8%)	31 16 (51.6%) 5 (16.1%) 5 (16.1%) 1 (3.2%) 0 (0%) 2 (6.5%) 1 (3.2%) 0 (0%) 0 (0%)
Registration			
BFR LSR	— —	71 (47.3%) 79 (52.6%)	3 (11.1%) 28 (88.9%)
Approach			
burr hole Twist drill	151 (85.3%) 26 (14.7%)	49 (29.2%) 119 (70.8%)	31 (100%)
Skin‐to‐skin (min)			
overall Burr hole Twist drill	60.4 ± 33.1 66.2 ± 32.1 26.5 ± 9.7	34.6 ± 34.5 55.5 ± 31.7 18.6 ± 9.4	64.6 ± 25.5 64.6 ± 25.5
Histology			
HGG LGG metastasis Lymphoma Others Non conclusive	89 (50.3%) 28 (15.8%) 5 (2.8%) 22 (12.4%) 32 (18.1%) 1 (0.6%)	79 (52.7%) 20 (13.3%) 4 (2.7%) 22 (6.7%) 23 (15.3%) 2 (1.3%)	13 (41.9%) 3 (9.7%) 0 (0%) 10 (32.3%) 5 (16.1%) 0 (0%)
Complications			
Symptomatic bleeding Permanent deficits Revision surgery	5 (2.8%) 4 (2.3%) 2 (1.1%)	4 (2.7%) 1 (0.7%) 2 (1.3%)	0 (0%) 0 (0%) 0 (0%)
Diagnostic yield	176 (99.4%)	148 (98.6%)	31 (100%)

Abbreviations: BFR: bone fiducial registration. LSR: laser surface registration. misc: miscellaneous.

^a^ incl. thalamus.

### 3.2 Surgical Approach (SA)

The reduction in STS in the *ROBOT* group was attributed to the selection of the surgical approach (*SA*). All *NAVIGATION cases and* 85.3% (151/177) of the *FRAME* cases were performed as conventional *BH* (Table 1). After the introduction of the robot, there was a significant shift to *TD* approaches (119/168). In fact, *TD* approaches have shorter *STS* than *BH* (20.2 ± 9.9 vs. 64.1 ± 31.3 min; *H* = 235.24, *p* < 0.001, Kruskal–Wallis) independent of the applied system. We applied an *ANOVA* to disentangle the effect of robotics (*GROUP*) and the *SA* on the *STS* time. There was a significant main effect of both *GROUP* (*F* = 3.38, *p* = 0.035) and *SA* (*F* = 112.83, *p* < 0.001) without any interaction (*F* = 0.16, *p* = 0.688), proving that the application of the robot reduces *STS* independent of the applied surgical approach. The simplification of the *SA* in the *ROBOT* group contributed to the fact that we applied more than one trajectory to completely sample the tumour in 18/150 (12%) cases.

### 3.3 Trajectory Characteristics

**FIGURE 2:**
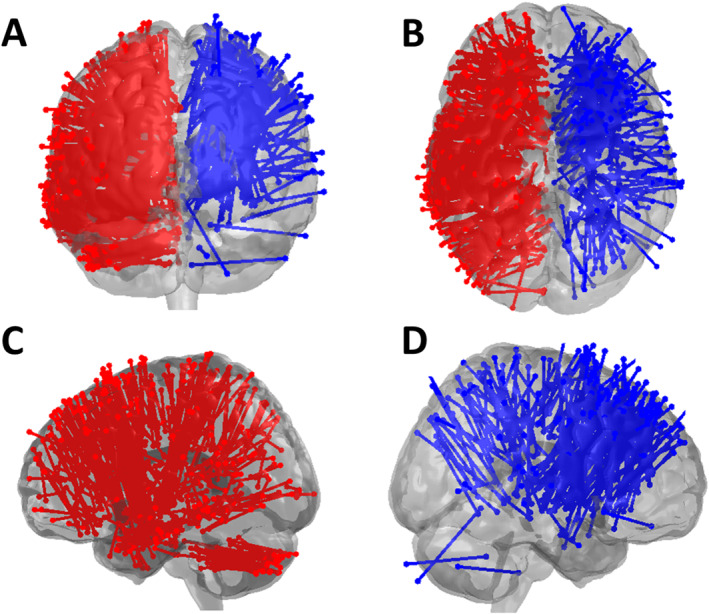
Overview over trajectories. The figure depicts all stereotactic trajectories in (A) anterior, (B) ventral, and lateral (C, D) views. There were less trajectories originating/targeting the very lateral aspect of the frontal and temporal lobe as well as the cerebellum in non‐robotic (*blue*) compared to robot‐assisted (*red*) surgeries. Robotic trajectories are mirrored to project to the left hemisphere, whereas non‐robotic trajectories are displayed at the right hemisphere.

**FIGURE 3:**
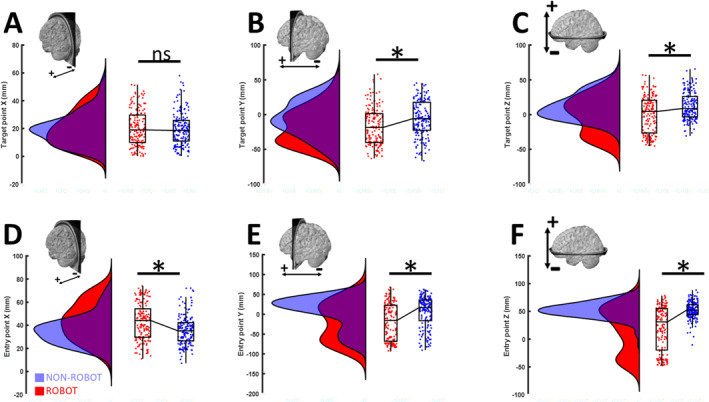
Entry and target point characteristics. After normalization of the individual MRI, we determined the target‐ (A, B, C) and entry (D, E, F) point coordinates (*X* *,Y,Z*) of non‐robotic (*blue*) and robot‐assisted trajectories (*red*) in the common *MNI* space. The introduction of the robotic technology increased the accessibility of target points in (B) the *Y*‐ (i.e., dorsal) and (C) *Z*‐axis (i.e., caudal). Furthermore, robotics liberated EP selection in (D) the *X*‐ (i.e., lateral), (E) *Y*‐ (i.e., dorsal) and (F) *Z*‐axis (i.e., caudal). Statistical significance (*p* < 0.05, Kruskal‐Wallis) is indicated by an asterisk (*).

The normalisation of the individual preoperative MR images enables the visualisation and analysis of all trajectories in the common *MNI* space (Figure 2). Notably, there were less trajectories originating or targeting the very lateral aspect of the brain as well as the temporal lobe and the cerebellum in the *NON‐ROBOT* surgeries compared with the *ROBOT* surgeries. Subsequently, we determined the coordinates of the EP and TP, the length of the trajectory (*TL*) as well as the trajectory angles about the y‐ (*α1*) and *x*‐axis (*α2*). This analysis confirmed that the introduction of the robotic technology increased the accessibility of target points on the Y‐ (i.e., dorsal) and *Z*‐axis (i.e., caudal). At the same time, the robotic assistance liberated *EP* selection in the X‐ (i.e., lateral), Y‐ (i.e., dorsal) and *Z*‐axis (i.e., caudal) (Figure 3).

**FIGURE 4:**
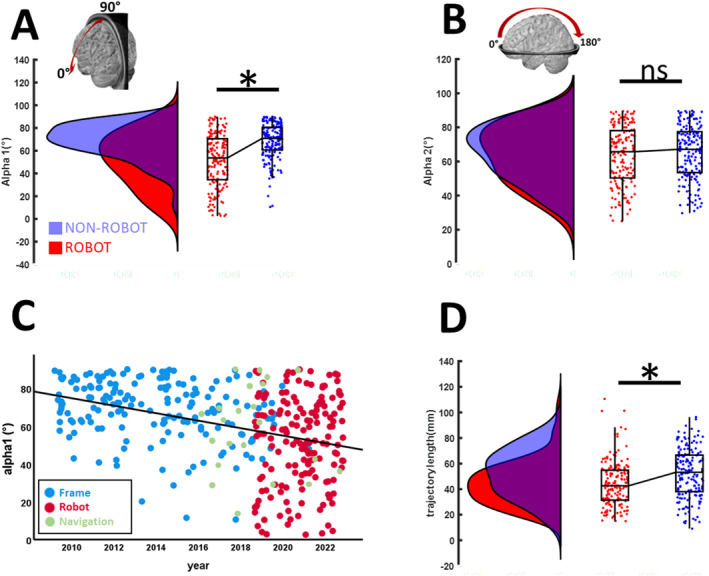
Trajectory characteristics. (A) There was a decrease in the trajectory angles about the *y*‐axis (α1) in robot‐assisted (*red*) compared to non‐robotic surgeries (*blue*), indicating an increased readiness to apply lateral trajectories. (B) In contrast, the trajectory angles about the *x*‐axis (α2) was unaffected. (C) α1‐angles reduced significantly within a few surgeries after the introduction of the robot. Notably, frame‐less neuronavigated biopsies were introduced earlier and did not present similar effects. (D) Trajectories applied with robotic assistance were significantly shorter than non‐robotic trajectories. Statistical significance (*p* < 0.05, Kruskal–Wallis) is indicated by an asterisk (*).

The application of the robot increased the readiness to apply lateral trajectories (i.e., reducing *α1*) while decreasing the length of the trajectory (Figure 4). *ROBOT* cases had smaller *α1* angles (51.6 ± 23.3°) compared to FRAME (68.4 ± 16.7°; *H* = 6.82, *p* < 0.001) and NEURONAVIGATION cases (57.4 ± 29.9°; *H* = 2.15, *p* = 0.032). Notably, there was no significant difference between FRAME and NAVIGATION cases (*H* = −1.16, *p* = 0.245). In contrast, there was no significant change in the *α2*‐angles of the trajectories when comparing FRAME, ROBOT and NAVIGATION cases (*H* = 0.985, *p* = 0.611). This modification of the stereotactic corridors occurred within a few surgeries after the introduction of the robot (Figure 4).

Separating trajectories according to the anatomical location of the *TP* determined that the robotic technology mainly affected trajectories to the frontal and the temporal lobes. For both locations, there was an increased application of lateral trajectories to the target (Table 2). In contrast, there was only a minor effect on trajectory planning to the parietal and occipital lobes as well as to the basal ganglia (incl. thalamus) or the insula. Notably, the access to the brainstem and the cerebellum was changed fundamentally. While these targets were hardly reachable with the stereotactic frame, the accessibility increased via robot‐assisted (*trans*‐) cerebellar trajectories (Table 2).

**TABLE 2:** Impact of target point location.

Target location	Non‐robot EP coordinates and trajectory parameter	Robot EP coordinates and trajectory parameter	*p*‐value
	*n* = 75	*n* = 38	
Frontal Lobe	**X: 29.7 ± 9.3 mm** Y: 20.0 ± 30.4 mm **Z: 29.7 ± 15.5 mm** **α1: 69.4 ± 14.6°** α2: 65.9 ± 15.8° **TL: 41.7 ± 15.1 mm**	**X: 37.3 ± 15.7 mm** Y: 20.0 ± 27.3 mm **Z: 45.2 ± 18.1 mm** **α1: 54.0 ± 20.9°** α2: 71.3 ± 14.6° **TL: 31.4 ± 15.4 mm**	***H* = 3.88; *p* = 0.049** *H* = 0.13; *p* = 0.723 ***H* = 5.65; *p* = 0.018** ***H* = 13.01; *p* < 0.001** *H* = 2.60; *p* = 0.107 ***H* = 12.95; *p* < 0.001**
	*n* = 27	*n* = 25	
Parietal Lobe	X: 36.7 ± 14.6 mm Y: −52.3 ± 19.5 mm Z: 58.2 ± 11.5 mm α1: 67.4 ± 17.2° α2: 68.0 ± 16.1° TL: 36.0 ± 14.4 mm	X: 39.7 ± 18.3 mm Y: −54.6 ± 16.8 mm Z: 47.4 ± 20.8 mm α1: 58.3 ± 25.7° α2: 68.2 ± 13.4° TL: 39.8 ± 10.4 mm	*H* = 0.08; *p* = 0.773 *H* = 0.56; *p* = 0.798 *H* = 2.10; *p* = 0.147 *H* = 1.05; *p* = 0.306 *H* = 0.01; *p* = 0.966 *H* = 0.56; *p* = 0.456
	*n* = 4	*n* = 5	
Occipital Lobe	X: 34.6 ± 6.5 mm Y: −76.5 ± 5.4 mm Z: 39.0 ± 6.4 mm α1: 76.7 ± 9.0° α2: 60.5 ± 9.4° TL: 40.9 ± 3.8 mm	X: 45.7 ± 18.1 mm Y: −67.1 ± 12.9 mm Z: 22.5 ± 31.1 mm α1: 43.0 ± 27.8° α2: 66.4 ± 15.1° TL: 42.3 ± 12.2 mm	*H* = 1.80; *p* = 0.180 *H* = 0.56; *p* = 0.456 *H* = 1.80; *p* = 0.180 *H* = 0.56; *p* = 0.456 *H* = 0.56; *p* = 0.456 *H* = 0.02; *p* = 0.881
	*n* = 21	*n* = 24	
Temporal Lobe	**X: 40.5 ± 18.5 mm** Y: −20.5 ± 45.4 mm **Z: 45.7 ± 23.5 mm** **α1: 68.7 ± 24.8°** α2: 67.4 ± 15.3° **TL: 67.1 ± 23.6 mm**	**X: 60.3 ± 14.4 mm** Y: −14.0 ± 22.2 mm **Z: 1.3 ± 29.2 mm** **α1: 29.7 ± 23.8°** α2: 76.1 ± 12.5° **TL: 39.4 ± 18.8 mm**	***H* = 8.01; *p* = 0.005** *H* = 0.09; *p* = 0.761 ***H* = 15.05; *p* < 0.001** ***H* = 12.68; *p* < 0.001** *H* = 3.03; *p* = 0.082 ***H* = 10.79; *p* < 0.001**
	*n* = 11	*n* = 7	
Insula	X: 44.7 ± 15.7 mm Y: 15.6 ± 25.4 mm Z: 45.6 ± 21.3 mm α1: 66.1 ± 25.2° α2: 72.0 ± 13.7° **TL: 50.8 ± 15.8 mm**	X: 52.8 ± 15.7 mm Y: 3.6 ± 22.3 mm Z: 30.6 ± 22.3 mm α1: 48.6 ± 31.7° α2: 66.4 ± 15.7° **TL: 38.4 ± 20.5 mm**	*H* = 0.46; *p* = 0.497 *H* = 1.50; *p* = 0.221 *H* = 1.73; *p* = 0.189 *H* = 1.50; *p* = 0.221 *H* = 0.91; *p* = 0.341 ***H* = 4.16; *p* = 0.041**
	*n* = 5	*n* = 8	
Brainstem	X: 36.2 ± 8.4 mm **Y: 20.5 ± 6.5 mm** **Z: 57.7 ± 1.1 mm** **α1: 70.9 ± 4.9°** **α2: 66.0 ± 2.6°** **TL: 83.7 ± 8.8 mm**	X: 43.7 ± 5.6 mm **Y: −74.4 ± 5.7 mm** **Z: −41.6 ± 4.7 mm** **α1: 53.4 ± 10.5°** **α2: 38.3 ± 9.6°** **TL: 57.3 ± 3.7 mm**	*H* = 1.85; *p* = 0.174 ***H* = 7.39; *p* = 0.007** ***H* = 7.39; *p* = 0.007** ***H* = 6.49; *p* = 0.011** ***H* = 7.39; *p* = 0.007** ***H* = 7.39; *p* = 0.007**
	*n* = 2	*n* = 28	
Cerebellum	X: 17.5 ± 10.6 mm Y: −70.5 ± 27.6 mm Z: −43.0 ± 10.8 mm α1: 44.3 ± 20.2° α2: 67.1 ± 24.6° TL: 53.0 ± 9.9 mm	X: 46.0 ± 5.4 mm Y: −77.7 ± 6.8 mm Z: −40.3 ± 4.1 mm α1: 44.3 ± 9.6° α2: 47.2 ± 10.6° TL: 48.5 ± 6.0 mm	— — — — — —
	*n* = 61	*n* = 30	
Basal Ganglia	X: 37.8 ± 11.0 mm Y: 20.0 ± 26.6 mm Z: 51.5 ± 10.6 mm α1: 68.0 ± 11.9° α2: 62.5 ± 16.3° **TL: 63.8 ± 10.5 mm**	X: 36.2 ± 12.6 mm Y: 23.0 ± 25.9 mm Z: 46.4 ± 16.3 mm α1: 66.3 ± 18.6° α2: 60.4 ± 14.7° **TL: 56.6 ± 13.6 mm**	*H* = 0.78; *p* = 0.378 *H* = 0.57; *p* = 0.449 *H* = 0.83; *p* = 0.362 *H* = 0.08; *p* = 0.784 *H* = 0.36; *p* = 0.549 ***H* = 9.60; *p* = 0.002**

*Note:* Trajectories were separated according to the anatomical location of the target point. Group differences in the (X,Y,Z) coordinates of the entry points, the length of the trajectory (TL) as well as the trajectory angles about the y‐ (α1) and *x*‐axis (α2) were evaluated. Notably, the cerebellum was avoided in non‐robotic surgery. Statistically significant values (*p* < 0.05, Kruskal–Wallis) are highlighted in bold.

Abbreviations: NON‐ROBOT: non‐robotic trajectories; ROBOT: robot‐assisted trajectories.

## 4 Discussion

This is one of the few studies examining trajectory planning for brain biopsies before (i.e., frame‐based) and after the introduction of frame‐less neuronavigation and robotic stereotaxy. Our analysis demonstrates that the application of a robot increased the allocation of *EP* and *TP* in stereotactic biopsies. While most frame‐based trajectories applied a high frontal or high parietal *EP*, a higher variability of the *EP* became evident in the *ROBOT* group. Namely, there was an increased readiness to apply more lateral trajectories while decreasing their length. This transformation of stereotactic corridors occurred within a few surgeries after the introduction of the robot. The introduction of frame‐less neuronavigated biopsies 2 years earlier did not have a comparable effect. At the same time, the duration and the invasiveness of the surgery decreased without affecting the diagnostic yield or the complication rate.

Frame‐based stereotaxy used to be the gold standard due to its proven accuracy. However, several studies have demonstrated that frameless techniques, including neuronavigation and robotic systems, are not inferior in terms of diagnostic yield and complication rates. For example, Dammers et al. (2008) [5] and Dhawan et al. (2019) [17] showed comparable safety and efficacy between frame‐based and frameless biopsies. More recently, Kesserwan et al. (2021) [18] and Ungar et al. (2022) [19] confirmed these findings in systematic reviews and meta‐analyses. In line, frameless techniques are widely—especially in U.S.‐based and high‐volume academic centres—accepted and increasingly preferred due to improved workflow, patient comfort, and comparable outcomes. Our data support the growing predominance of frameless stereotaxy over frame‐based techniques in the domain of brain biopsy. Frameless stereotaxy maintained the safety and diagnostic integrity of the procedure. The use of robotic assistance also introduced new degrees of freedom in trajectory selection. Compared to classic neuronavigation systems, the robotic platform offered improved stability, guidance precision, and reproducibility, particularly for twist drill approaches that are traditionally less favoured in frame‐based techniques due to skiving risk. Nevertheless, frame‐based stereotaxy is still applied—especially in E.U.‐based centres—for functional procedures (i.e., for Deep Brain Stimulation of the subthalamic nucleus in Parkinsons' disease) and for biopsies of small or poorly defined lesions in eloquent or highly critical areas, where even minimal offset can have significant clinical consequences [18, 20, 21].

Our results confirm previous literature showing high *DY* (approx. 90%–95% [4, 5, 22]) and a low morbidity (approx. 0%–8.6% [23]) for both frame‐based and frame‐less techniques. Marcus et al. performed a meta‐analysis on 328 robot‐assisted brain biopsies showing an overall risk of serious complications of less than 1% [24]. The same analysis indicated a *DY* of 95% after robot‐assisted brain biopsy. At the same time, our results demonstrate that the application of the robot significantly decreases the *STS* of intracranial biopsies compared with conventional frame‐based and navigation techniques. Furthermore, the presented *STS* durations are shorter than other reports on frame‐based [4, 17] or robot‐assisted biopsies [25, 26, 27, 28, 29, 30, 31]. We attribute this observation to a constant learning curve and to the modification of the surgical approach [30, 32, 33]. In the present analysis, we observed a shift from burr hole to twist drill craniotomies after the introduction of the robot. Twist drill approaches are usually avoided in stereotaxy as there is the risk of slipping along the skull in tangential trajectories [34]. In conventional frame‐based and navigation‐based brain biopsies, this dogma is widespread and explained by the poor guidance of the twist drill. Due to the centre‐of‐arc principle of conventional stereotactic frames, the distance between the guiding tube and the skull is immutable and is determined by the positioning of the frame on the skull and the depth of the target point. In the present robotic system, this distance is adaptable with a very stable guidance of the drill. In contrast, most navigation‐based techniques provide only limited stability.

There are several current reports suggesting different twist drill techniques for brain biopsies [19, 35, 36]. The advantage of the (ROSA) robot in comparison to neuronavigation (e.g., VarioGuide) is the stability of the guidance and the possibility to actively change the distance to the skull on‐the‐fly during surgery [18, 37, 38]. This move provides a very good guidance of the drill minimising the risk of inaccuracy. Our own group has shown no accuracy differences between orthogonal and oblique trajectories in robot‐assisted stereotaxy and no differences to conventional frame‐based surgeries [39]. This is in line with the analysis of Rollo et al., who proved in a series of 150 robot‐assisted procedures that oblique trajectories do not entail a higher complication risk [40]. Another advantage of twist drill approaches is the neglectable CSF leakage preventing brain shift. Consequently, the positioning of the patient is simplified and the craniotomy is not necessarily the highest point on the cranium.

Our analysis indicates that there was an increased readiness in robot‐assisted stereotaxy to apply more lateral trajectories while decreasing their length. In contrast, there was only a minor effect on trajectory targeting the parietal lobe, the basal ganglia (incl. thalamus) or the insula. Trajectories to these targets are additionally limited by eloquent cortex or vascular structures, e.g., speech‐related cortex within the left parietal lobe. Additionally, the Sylvian fissure with its vulnerable vessels insulates the basal ganglia and the insula predefining the stereotactic corridors. Notably, the robotic technology facilitated trajectories to the cerebellum and the transcerebellar access to the brainstem [10, 41, 42, 43], which are usually avoided in conventional frame‐based stereotaxy [43, 44, 45]. Prior to the introduction of the robot, brainstem lesions were target using a transfrontal approach, whereas cerebellar lesions were mainly probed by open surgery.

However, there is still missing evidence of a clinical benefit or surgical superiority and worse cost‐efficiency of robotic frame‐less solutions [29, 31, 46, 47]. A substantial reduction in operating time has been described for stereotactic surgeries involving several trajectories, that is, stereoelectroencephalography [39, 48, 49, 50]. For stereotactic procedures with single trajectories (i.e., biopsies), time saving by the application of robotics is less but still consistent and noteworthy [29, 31]. In particular, the registration process necessitates additional time, which impairs the time efficiency of neuronavigation and robot‐assisted approaches. It is well documented, that workflow and the overall case time are significantly affected by the applied registration technique (e.g., BFR or LSR) [29, 31]. However, the present study documents a significant STS acceleration and reduction of invasiveness in stereotactic biopsies by robotics. Together with the faster recovery and discharge of the patient, robotics might achieve cost‐efficiency even for intracranial biopsies. In addition, our study shows how the robotic technology expands stereotactic corridors and facilitates trajectories which were hardly applicable in conventional frame‐based stereotaxy. It is noteworthy, that there are other innovative technical solutions for establishing stereotactic access to the brain (e.g., Starfix or ClearPoint), which might also enable free trajectory selection. However, so far, there are only limited data on the time efficiency of these solutions [51].

## 5 Conclusion

In summary, our findings support a growing body of global literature suggesting that frame‐less (including robotic) stereotactic techniques are safe and effective. Our findings suggest that robotic systems combine the flexibility of frameless navigation with improved procedural accuracy and efficiency. The added flexibility, efficiency, and access provided by robotics may offer a clinical benefit in certain contexts. These benefits may help justify the cost and learning curve associated with robotic implementation, particularly when trajectory diversity or surgical workflow optimization is critical.

## Author Contributions

Study conception and design: Georgios Naros. Data acquisition: Eliane Weinbrenner, Mykola Gorbachuk, Georgios Naros, Kathrin Machetanz and Florian Grimm. Data analysis: Georgios Naros and Eliane Weinbrenner. Data interpretation: Georgios Naros and Eliane Weinbrenner. Statistical analysis: Eliane Weinbrenner and Georgios Naros. Writing of the first draft: Georgios Naros and Eliane Weinbrenner. Review of the final manuscript: Georgios Naros, Kathrin Machetanz, Mykola Gorbachuk, Sophie S. Wang, Florian Grimm and Marcos Tatagiba.

## Ethics Statement

The study was approved by the local ethics committee of the Medical Faculty of the Eberhard Karls University Tuebingen.

## Consent

All participants gave written informed consent.

## Conflicts of Interest

The authors declare that the research was conducted in the absence of any commercial or financial relationships that could be construed as a potential conflict of interest.

## Data Availability

The data that support the findings of this study are available from the corresponding author upon reasonable request.

## References

[1] E. A. Spiegel , H. T. Wycis , M. Marks , and A. J. Lee , “Stereotaxic Apparatus for Operations on the Human Brain,” Science 106, no. 2754 (1947): 349–350, 10.1126/science.106.2754.349 17777432

[2] A. M. Lozano , P. L. Gildenberg , and R. R. Tasker , Textbook of Stereotactic and Functional Neurosurgery, 1 (Springer Science & Business Media, 2009).

[3] N. Pouratian and S. A. Sheth , Stereotactic and Functional Neurosurgery (Springer Nature, 2020), 10.1007/978-3-030-34906-6.

[4] Y. Lu , C. Yeung , A. Radmanesh , R. Wiemann , P. M. Black , and A. J. Golby , “Comparative Effectiveness of Frame‐Based, Frameless, and Intraoperative Magnetic Resonance Imaging‐Guided Brain Biopsy Techniques,” World Neurosurgery 83, no. 3 (2015): 261–268, 10.1016/j.wneu.2014.07.043. 25088233 PMC4450019

[5] R. Dammers , I. K. Haitsma , J. W. Schouten , J. M. Kros , C. J. J. Avezaat , and A. J. P. E. Vincent , “Safety and Efficacy of Frameless and Frame‐Based Intracranial Biopsy Techniques,” Acta Neurochirurgica 150, no. 1 (2008): 23–29: Published online, 10.1007/s00701-007-1473-x. 18172567

[6] M. Harary and G. R. Cosgrove , “Jean Talairach: A Cerebral Cartographer,” Neurosurgical Focus 47, no. 3 (2019): E12, 10.3171/2019.6.FOCUS19320. 31473671

[7] W. J. Elias , C. A. Sansur , and R. C. Frysinger , “Sulcal and Ventricular Trajectories in Stereotactic Surgery,” Journal of Neurosurgery 110, no. 2 (2009): 201–207, 10.3171/2008.7.17625. 18821828

[8] L. Zrinzo , A. L. J. Van Hulzen , A. A. Gorgulho , et al., “Avoiding the Ventricle: A Simple Step to Improve Accuracy of Anatomical Targeting During Deep Brain Stimulation ‐ Clinical Article,” Journal of Neurosurgery 110, no. 6 (2009): 1283–1290, 10.3171/2008.12.JNS08885. 19301961

[9] H. Joswig , A. G. Parrent , K. W. MacDougall , and D. A. Steven , “Prohibited Arc Angles During Leksell Frame‐Based Stereotaxy,” World Neurosurgery 112 (2018): 123–125, 10.1016/J.WNEU.2018.01.068. 29391299

[10] K. Machetanz , F. Grimm , S. Wang , et al., “Rediscovery of the Transcerebellar Approach: Improving the Risk‐Benefit Ratio in Robot‐Assisted Brainstem Biopsies,” Neurosurgical Focus 52, no. 1 (2022): E12, 10.3171/2021.10.FOCUS21359. 34973665

[11] W. J. Elias , K. M. Fu , and R. C. Frysinger , “Cortical and Subcortical Brain Shift During Stereotactic Procedures,” Journal of Neurosurgery 107, no. 5 (2007): 983–988, 10.3171/JNS-07/11/0983. 17977271

[12] E. A. Petersen , E. M. Holl , I. Martinez‐Torres , et al., “Minimizing Brain Shift in Stereotactic Functional Neurosurgery,” Neurosurgery 67, no. S1 (2010): ons213–ons221, 10.1227/01.NEU.0000380991.23444.08. 20679927

[13] F. Grimm , G. Naros , A. Gutenberg , N. Keric , A. Giese , and A. Gharabaghi , “Blurring the Boundaries Between Frame‐Based and Frameless Stereotaxy: Feasibility Study for Brain Biopsies Performed With the Use of a Head‐Mounted Robot,” Journal of Neurosurgery 123, no. 3 (2015): 737–742, 10.3171/2014.12.JNS141781. 26067616

[14] E. von Elm , D. G. Altman , M. Egger , S. J. Pocock , P. C. Gøtzsche , and J. P. Vandenbroucke , “The Strengthening the Reporting of Observational Studies in Epidemiology (STROBE) Statement: Guidelines for Reporting Observational Studies,” Lancet 370, no. 9596 (2007): 1453–1457, 10.1016/S0140-6736(07)61602-X. 18064739

[15] K. J. Friston , J. T. Ashburner , S. J. Kiebel , T. E. Nichols , and W. D. Penny , Statistical Parametric Mapping: The Analysis of Functional Brain Images, (Elsevier, 2007), 10.1016/B978-0-12-372560-8.X5000-1.

[16] N. Tzourio‐Mazoyer , B. Landeau , D. Papathanassiou , et al., “Automated Anatomical Labeling of Activations in SPM Using a Macroscopic Anatomical Parcellation of the MNI MRI Single‐Subject Brain,” Neuroimage 15, no. 1 (2002): 273–289, 10.1006/nimg.2001.0978. 11771995

[17] S. Dhawan , Y. He , J. Bartek , A. A. Alattar , and C. C. Chen , “Comparison of Frame‐Based Versus Frameless Intracranial Stereotactic Biopsy: Systematic Review and Meta‐Analysis,” World Neurosurgery 127 (2019): 607–616.e4: Published online, 10.1016/j.wneu.2019.04.016. 30974279

[18] M. A. Kesserwan , H. Shakil , M. Lannon , et al., “Frame‐Based Versus Frameless Stereotactic Brain Biopsies: A Systematic Review and Meta‐Analysis,” Surgical Neurology International 12 (2021): 52, 10.25259/SNI_824_2020. 33654555 PMC7911151

[19] L. Ungar , O. Nachum , Z. Zibly , et al., “Comparison of Frame‐Based Versus Frameless Image‐Guided Intracranial Stereotactic Brain Biopsy: A Retrospective Analysis of Safety and Efficacy,” World Neurosurgery 164 (2022): e1–e7, 10.1016/j.wneu.2021.07.063. 34332151

[20] C. Neudorfer , S. Hunsche , M. Hellmich , F. El Majdoub , and M. Maarouf , “Comparative Study of Robot‐Assisted Versus Conventional Frame‐Based Deep Brain Stimulation Stereotactic Neurosurgery,” Stereotactic and Functional Neurosurgery 96, no. 5 (2018): 327–334, 10.1159/000494736. 30481770

[21] L. R. Philipp , C. M. Matias , S. Thalheimer , S. H. Mehta , A. Sharan , and C. Wu , “Robot‐Assisted Stereotaxy Reduces Target Error: A Meta‐Analysis and Meta‐Regression of 6056 Trajectories,” Neurosurgery 88, no. 2 (2021): 222–233, 10.1093/NEUROS/NYAA428. 33045739

[22] S. Katzendobler , A. Do , J. Weller , et al., “Diagnostic Yield and Complication Rate of Stereotactic Biopsies in Precision Medicine of Gliomas,” Frontiers in Neurology 13 (2022): 513, 10.3389/FNEUR.2022.822362/BIBTEX. PMC9005817 35432168

[23] R. Grossman , S. Sadetzki , R. Spiegelmann , and Z. Ram , “Haemorrhagic Complications and the Incidence of Asymptomatic Bleeding Associated With Stereotactic Brain Biopsies,” Acta Neurochirurgica 147, no. 6 (2005): 627–631: Published online, 10.1007/s00701-005-0495-5. 15821863

[24] H. J. Marcus , V. N. Vakharia , S. Ourselin , J. Duncan , M. Tisdall , and K. Aquilina , “Robot‐Assisted Stereotactic Brain Biopsy: Systematic Review and Bibliometric Analysis,” Child's Nervous System 34, no. 7 (2018): 1299–1309, 10.1007/S00381-018-3821-Y. PMC5996011 29744625

[25] M. Lefranc , C. Capel , A. S. Pruvot‐Occean , et al., “Frameless Robotic Stereotactic Biopsies: A Consecutive Series of 100 Cases,” Journal of Neurosurgery 122, no. 2 (2015): 342–352, 10.3171/2014.9.JNS14107. 25380111

[26] M. Lefranc , C. Capel , A. S. Pruvot , et al., “The Impact of the Reference Imaging Modality, Registration Method and Intraoperative Flat‐Panel Computed Tomography on the Accuracy of the ROSA® Stereotactic Robot,” Stereotactic and Functional Neurosurgery 92, no. 4 (2014): 242–250: Published online, 10.1159/000362936. 25170634

[27] A. De Benedictis , A. Trezza , A. Carai , et al., “Robot‐Assisted Procedures in Pediatric Neurosurgery,” Neurosurgical Focus 42, no. 5 (2017): E7: Published online, 10.3171/2017.2.FOCUS16579. 28463617

[28] L. Terrier , V. Gilard , F. Marguet , M. Fontanilles , and S. Derrey , “Stereotactic Brain Biopsy: Evaluation of Robot‐Assisted Procedure in 60 Patients,” Acta Neurochirurgica 161, no. 3 (2019): 545–552: Published online, 10.1007/s00701-019-03808-5. 30675655

[29] K. Bekelis , T. A. Radwan , A. Desai , and D. W. Roberts , “Frameless Robotically Targeted Stereotactic Brain Biopsy: Feasibility, Diagnostic Yield, and Safety,” Journal of Neurosurgery 116, no. 5 (2012): 1002–1006, 10.3171/2012.1.JNS111746. 22404667

[30] K. Machetanz , F. Grimm , M. Schuhmann , M. Tatagiba , A. Gharabaghi , and G. Naros , “Time Efficiency in Stereotactic Robot‐Assisted Surgery: An Appraisal of the Surgical Procedure and Surgeon’s Learning Curve,” Stereotactic and Functional Neurosurgery 99, no. 1 (2021): 25–33, 10.1159/000510107. 33017833

[31] G. Naros , K. Machetanz , F. Grimm , F. Roser , A. Gharabaghi , and M. Tatagiba , “Framed and Non‐Framed Robotics in Neurosurgery: A 10‐Year Single‐Center Experience,” International Journal of Medical Robotics and Computer Assisted Surgery 17, no. 5 (2021), 10.1002/rcs.2282. 34030218

[32] A. Khan , J. E. Meyers , I. Siasios , and J. Pollina , “Next‐Generation Robotic Spine Surgery: First Report on Feasibility, Safety, and Learning Curve,” Operative Neurosurgery 17, no. 1 (2019): 61–68, 10.1093/ons/opy280. 30247684

[33] R. A. McGovern , R. S. Butler , J. Bena , and J. Gonzalez‐Martinez , “Incorporating New Technology into a Surgical Technique: The Learning Curve of a Single Surgeon’s Stereo‐Electroencephalography Experience,” Clinical Neurosurgery 86, no. 3 (2020): E281–E289, 10.1093/neuros/nyz498. 31813973

[34] J. C. Iordanou , D. Camara , S. Ghatan , and F. Panov , “Approach Angle Affects Accuracy in Robotic Stereoelectroencephalography Lead Placement,” World Neurosurgery 128 (2019): e322–e328, 10.1016/j.wneu.2019.04.143. 31028981

[35] S. Al‐Saiari , A. A. Farag , K. Al Orabi , M. Abdoh , and H. Kheshaifati , “A Simple Modified Technique for Frameless Brain Lesion Biopsy,” Cureus 12, no. 12 (2020), 10.7759/cureus.12002. PMC7797436 33457113

[36] G. Minchev , G. Kronreif , W. Ptacek , et al., “A Novel Robot‐Guided Minimally Invasive Technique for Brain Tumor Biopsies,” Journal of Neurosurgery 132, no. 1 (2020): 150–158, 10.3171/2018.8.JNS182096. 30660122

[37] J. D. Sharma , K. K. Seunarine , M. Z. Tahir , and M. M. Tisdall , “Accuracy of Robot‐Assisted Versus Optical Frameless Navigated Stereoelectroencephalography Electrode Placement in Children,” Journal of Neurosurgery: Pediatrics 23, no. 3 (2019): 297–302, 10.3171/2018.10.PEDS18227. 30611155

[38] F. Girgis , E. Ovruchesky , J. Kennedy , M. Seyal , K. Shahlaie , and I. Saez , “Superior Accuracy and Precision of SEEG Electrode Insertion With Frame‐Based vs. Frameless Stereotaxy Methods,” Acta Neurochirurgica 162, no. 10 (2020): 2527–2532, 10.1007/S00701-020-04427-1/FIGURES/3. 32458403

[39] K. Machetanz , F. Grimm , T. V. Wuttke , et al., “Frame‐Based and Robot‐Assisted Insular Stereo‐Electroencephalography via an Anterior or Posterior Oblique Approach,” Journal of Neurosurgery 135, no. 5 (2021): 1477–1486, 10.3171/2020.10.JNS201843. 33930861

[40] P. S. Rollo , M. J. Rollo , P. Zhu , O. Woolnough , and N. Tandon , “Oblique Trajectory Angles in Robotic Stereo‐Electroencephalography,” Journal of Neurosurgery 135, no. 1 (2020): 245–254, 10.3171/2020.5.JNS20975. 32796145

[41] A. Carai , A. Mastronuzzi , A. De Benedictis , et al., “Robot‐Assisted Stereotactic Biopsy of Diffuse Intrinsic Pontine Glioma: A Single‐Center Experience,” World Neurosurgery 101 (2017): 584–588: Published online, 10.1016/j.wneu.2017.02.088. 28254596

[42] M. Gupta , T. M. Chan , D. R. Santiago‐Dieppa , et al., “Robot‐Assisted Stereotactic Biopsy of Pediatric Brainstem and Thalamic Lesions,” Journal of Neurosurgery: Pediatrics 27, no. 3 (2020): 1–8: Published online, 10.3171/2020.7.peds20373. 33361479

[43] M. Dellaretti , N. Reyns , G. Touzet , et al., “Stereotactic Biopsy for Brainstem Tumors: Comparison of Transcerebellar With Transfrontal Approach,” Stereotactic and Functional Neurosurgery 90, no. 2 (2012): 79–83: Published online, 10.1159/000335502. 22286495

[44] P. Kickingereder , P. Willeit , T. Simon , and M. I. Ruge , “Diagnostic Value and Safety of Stereotactic Biopsy for Brainstem Tumors: A Systematic Review and Meta‐Analysis of 1480 Cases,” Neurosurgery 72, no. 6 (2013): 873–882: Published online, 10.1227/NEU.0b013e31828bf445. 23426149

[45] J. Steck and W. A. Friedman , “Stereotactic Biopsy of Brainstem Mass Lesions,” Surgical Neurology 43, no. 6 (1995): 563–568: Published online, 10.1016/0090-3019(95)00156-5. 7482235

[46] B. Fiani , S. A. Quadri , M. Farooqui , et al., “Impact of Robot‐Assisted Spine Surgery on Health Care Quality and Neurosurgical Economics: A Systemic Review,” Neurosurgical Review 43, no. 1 (2020): 17–25, 10.1007/s10143-018-0971-z. 29611081

[47] J. A. Smith , J. Jivraj , R. Wong , and V. Yang , “30 Years of Neurosurgical Robots: Review and Trends for Manipulators and Associated Navigational Systems,” Annals of Biomedical Engineering 44, no. 4 (2016): 836–846, 10.1007/s10439-015-1475-4. 26467553

[48] P. Bourdillon , C. E. Châtillon , A. Moles , et al., “Effective Accuracy of Stereoelectroencephalography: Robotic 3D Versus Talairach Orthogonal Approaches,” Journal of Neurosurgery 131, no. 6 (2019): 1938–1946: Published online, 10.3171/2018.7.jns181164. 30544338

[49] T. J. Abel , R. V. Osorio , R. Amorim‐Leite , et al., “Frameless Robot‐Assisted Stereoelectroencephalography in Children: Technical Aspects and Comparison With Talairach Frame Technique,” Journal of Neurosurgery: Pediatrics 22, no. 1 (2018): 37–46: Published online, 10.3171/2018.1.PEDS17435. 29676681

[50] J. González‐Martínez , J. Bulacio , S. Thompson , et al., “Technique, Results, and Complications Related to Robot‐Assisted Stereoelectroencephalography,” Neurosurgery 78, no. 2 (2016): 169–180, 10.1227/NEU.0000000000001034. 26418870

[51] B. R. Taha , C. R. Osswald , M. Rabon , et al., “Learning Curve Associated With ClearPoint Neuronavigation System: A Case Series,” World Neurosurgery X 13 (2021): 100115, 10.1016/J.WNSX.2021.100115. 35028557 PMC8739880

